# Unconventional localization of PAI-1 in PML bodies: A possible link with cellular growth of endothelial cells

**DOI:** 10.1016/j.bbrep.2024.101793

**Published:** 2024-07-26

**Authors:** Pragya Gehlot, Daniela Brünnert, Vibha Kaushik, Arpana Yadav, Saloni Bage, Kritika Gaur, Mahesh Saini, Jens Ehrhardt, Gowrang Kasaba Manjunath, Abhishek Kumar, Neena Kasliwal, Ajay Kumar Sharma, Marek Zygmunt, Pankaj Goyal

**Affiliations:** a Department of Biotechnology, School of Life Sciences, Central University of Rajasthan, Bandarsindri, Kishangarh, 305 817, Rajasthan, India; b University Hospital of Würzburg, Department of Obstetrics and Gynecology, Josef-Schneider-Str. 4, D-97080, Würzburg, Germany; c Department of Obstetrics and Gynecology, University of Greifswald, Ferdinand-Sauerbruchstrasse, D-17489, Greifswald, Germany; d Manipal Academy of Higher Education (MAHE), Manipal, 576104, Karnataka, India; e Institute of Bioinformatics, International Technology Park, Bangalore, 560066, Karnataka, India; f Department of Pathology, J.L.N. Medical College, Ajmer, 305001, Rajasthan, India; g Department of Obstetrics and Gynecology, J.L.N. Medical College, Ajmer, 305001, Rajasthan, India

**Keywords:** PAI-1, PML, Endothelial cells, Senescence, Serpin E1, CRM1

## Abstract

Plasminogen activator inhibitor-1 (PAI-1/Serpin E1) is classically known for its antifibrinolytic activity via inhibiting uPA and tPA of the fibrinolytic pathway. PAI-1 has a paradoxical role in tumor progression, and its molecular functions are poorly understood. PAI-1 is a widely accepted secretory protease inhibitor, however, a study suggested the localization of PAI-1 in the cytoplasm and the nucleus. Besides the plethora of its biological functions as a secretory protein, intracellular localization, and functions of PAI-1 remain unexplored at the molecular level. In this study, using various *in silico* approaches, we showed that PAI-1 possesses a nuclear export signal. Using the CRM1-specific inhibitor leptomycin B, we demonstrated that PAI-1 has a functional CRM1-dependent NES, indicating the possibility of its nuclear localization. Further, we confirm that PAI-1 is localized in the nucleus of endothelial cells using fluorescence microscopy and immunoprecipitation. Notably, we identified an unconventional distribution of PAI-1 in the PML bodies of the nucleus of normal endothelial cells, while the protein was restricted in the cytoplasm of slow-growing cells. The data showed that the localization of PAI-1 in PML bodies is highly correlated with the growth potential of endothelial cells. This conditional nucleocytoplasmic shuttling of PAI-1 during the aging of cells could impart a strong link to its age-related functions and tumor progression. Together, this study identifies the novel behavior of PAI-1 that might be linked with cell aging and may be able to unveil the elusive role of PAI-1 in tumor progression.

## Highlights

• PAI-1 has a functional CRM1-dependent nuclear export signal.
• PAI-1 is localized in the PML bodies of normal endothelial cells.
• PAI-1 in PML bodies is correlated with the growth potential of endothelial cells.
• Nucleocytoplasmic shuttling of PAI-1 could impart a strong link to tumor growth.

## Graphical abstract

**Figure:**
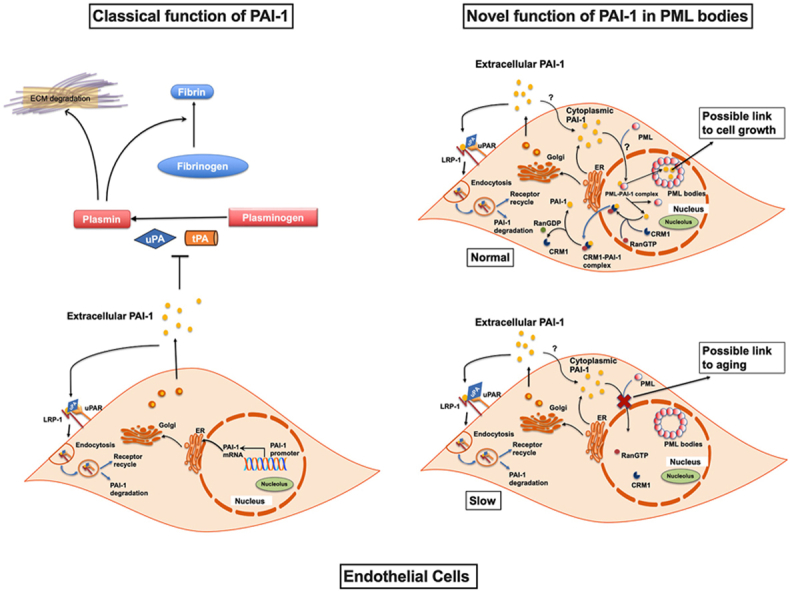
Extracellular PAI-1 is known for its antifibrinolytic activity (left panel). We identified an unconventional distribution of PAI-1 in the PML bodies, which is highly correlated with the growth potential of endothelial cells. Together, this study identifies the novel behavior of PAI-1 that might be linked with cell aging and may be able to unveil the elusive role of PAI-1 in tumor progression.

## 1 Introduction

Plasminogen activator inhibitor-1 (PAI-1)/Serpin E1, a multifarious secretory protease inhibitor, belongs to the group V3 of the Serpin superfamily [1]. The classical function of PAI-1 is to regulate fibrinolysis by inhibiting two potent plasminogen activators, tPA and uPA, that convert plasminogen into plasmin [2]. PAI-1 is present as a minor protein in plasma, but, under various disease conditions, such as cancer and diabetes, its expression was highly upregulated not only in plasma but also in the extracellular matrix (ECM) [3,4]. PAI-1 is secreted in the ECM and inhibits its degradation, thereby regulating the accumulation of ECM, suggesting its inhibitory effects on cell migration, proliferation, and invasion [5,6]. PAI-1 also binds with vitronectin and inhibits the vitronectin-integrin V3 interaction, affecting a cascade of cell functions, such as cell adhesion [5], migration [5,7], proliferation [8], and apoptosis [6], depicting the anti-tumor roles of PAI-1.

In contrast, tumor growth and vascularization were reduced in PAI-1 knockout mice [9]. PAI-1 promotes tumor growth by influencing the progression of the G0/G1 phase cell cycle [10]. Increased levels of PAI-1 were indicated to predict poor clinical outcomes in various types of cancers, including gastric [11,12], breast [13], ovarian [14,15], and lung cancer [16,17], showing the paradoxical functions of PAI-1 in tumor progression.

Moreover, the expression levels of PAI-1 mRNA and protein get elevated in chronological and stress-induced senescence [18], suggesting its role in various age-related complications. PAI-1 is also a crucial component of the senescence-related secretome [19], and a direct mediator and biomarker of cellular senescence [20]. PAI-1-deficient fibroblasts are resistant to senescence and can proliferate longer than the wild type, suggesting PAI-1 to be a positive regulator of senescence [21].

Apart from these, Promyelocytic Leukemia Protein-Nuclear Bodies (PML-NBs), mainly formed by PML protein, are critical regulators of senescence [22]. Overexpression of PML protein induced senescence in mouse and human fibroblasts in a p53-dependent manner [23]. The splice variant IV of PML gets overexpressed and interacts with the tumor suppressor protein, ARF, a key p53 regulator, and promotes post-translational modifications, such as SUMOylation of p53, leading to its stabilization and activation, which ultimately triggers senescence [24].

PAI-1 interacts with proteasomes in TNF-α and LPS-activated human endothelial cell line EA.hy926 [25]. Interestingly, previous studies showed a diffused localization of PAI-1 in the cytoplasm, Golgi bodies, perinuclear region, and the nucleus [[15], [25],^26^]. Besides the plethora of its biological functions as a secretory protein, intracellular localization, and functions of PAI-1 remain unexplored. In this study, we report that PAI-1 possesses a CRM1-dependent nuclear export signal (NES). Regarding this, a question arose whether PAI-1 gets localized in the nucleus, and if yes, is it linked with PML bodies in the nucleus? In this direction, we found PAI-1 localization in PML bodies in the nucleus of normal endothelial cells, while the protein was restricted in the cytoplasm of slow-growing cells. This conditional nucleocytoplasmic shuffling of PAI-1 during the aging of cells could impart a strong link to its age-related functions.

## 2 Materials and methods

### 2.1 Materials

Hoechst 33258, Alexa Fluor IgG 488 goat *anti*-mouse, Alexa Fluor IgG 555 goat *anti*-mouse, and Alexa Fluor 555 goat *anti*-rabbit were procured from Thermo Fisher Scientific. Endothelial growth medium was purchased from Promo Cell and Thermo Fisher Scientific. Tubulin antibody (E7) was procured from the Developmental Studies Hybridoma Bank (DSHB). Leptomycin B (LMB) was from Selleckchem, and monoclonal mouse PAI-1 (C-09) antibody and rabbit PML antibody were purchased from Santa Cruz Biotechnology. Mouse IgG2b kappa antibodies were from BD Pharmingen. The rabbit IgG was purchased from Cell Signaling Technology. Secondary antibodies, goat anti-rabbit 680RD antibody and goat *anti*-mouse 800CW antibody were purchased from LI-COR Biosciences.

### 2.2 Cell culture

The human umbilical vein endothelial cells (HUVECs) were isolated from the umbilical cords after obtaining pre-informed and written consent from the Institutional Ethical Committee at the J.L.N. Medical College, Ajmer, India, and the University of Greifswald, Germany. These cells were plated onto 75 cm^2^ rat tail collagen-coated tissue culture flasks in a complete endothelial growth medium and were grown as described earlier [27].

### 2.3 Immunofluorescence staining

HUVECs were grown on collagen-coated 8-well Lab-Tek 2 chambers until they reached 80–90 % confluence. HUVECs were treated with 10 ng/ml LMB for 2 h to inhibit CRM1, and MeOH served as control. Cells were then fixed for 10 min with 3.7 % PFA, permeabilized for 20 min using 0.2 % Triton-X100 in PBS/T, and stained with 10 μg/ml Hoechst 33258. Non-specific binding was blocked with 2 % fatty acid-free BSA in PBS/T for 40 min, followed by an incubation of the primary antibodies in 2 % fatty acid-free BSA in PBS/T for 60 min. Secondary antibody incubation was performed using 2 % fatty acid-free BSA in PBS/T for 45 min.

The primary antibodies were used at the dilutions: PAI-1 (C-09) antibody (1:75), PML antibody (1:150), IgG2b kappa antibodies (1:100), and rabbit IgG (1:150). The secondary antibodies were used at a dilution of 1:200. Images were captured using a Zeiss fluorescence microscope with Zeiss Axio Vision 4.8 software for data analysis.

### 2.4 Immunoprecipitation and Western blotting

Immunoprecipitation from nuclear lysate was performed using the Nuclear Co-IP-Kit (Active motif, Inc., USA) according to the manufacturer's instructions. The nuclear lysates from HUVECs were incubated with 3 μg PAI-1 (C-09) antibody overnight. The next day, 25 μl protein G microbeads were added to the suspension and incubated for 1 h. After washing the beads, samples were prepared using 2X Laemmli buffer.

The Co-IP samples were subjected to 12 % SDS-gel electrophoresis, and the proteins were then transferred to a PVDF membrane using the Mini Trans-Blot electrophoresis cell. The membrane was blocked with Odyssey blocking buffer and incubated with primary and secondary antibodies. PAI-1 mouse (C-09) and PML rabbit and cytoplasmic marker tubulin (E7) antibodies were used at dilutions of 1:1000,1:1,500, and 1:1,000, respectively. The secondary antibodies were used at a dilution of 1:10,000. The protein signal was detected using the Odyssey Infrared Imager (LI-COR Biosciences).

### 2.5 *In silico* motif analysis

PAI-1 protein sequence (NP_000593.1) was subjected to the *in*-silico motif analysis tools; ELM server, and LocNES tool. NES (cut-off score >0.1) were selected from the LocNES predicted motifs for further analyses. The PAI-1 sequence was manually analyzed using the NES prediction consensus patterns [28].

### 2.6 Assessment of the solvent accessibility of NES motif

The solvent-accessible area of NES was assessed using BIOVIA Discovery Studio 2020 Client Visualizer. The percentage surface accessibility score (PSA) was calculated with a probe radius of 1.4 Å and 240 grid points. Motifs showing a PSA score >25 were considered surface-accessible.

## 3 Results

### 3.1 PAI-1 has a potential CRM1-specific nuclear export signal but not a nuclear localization signal

**Fig. 1:**
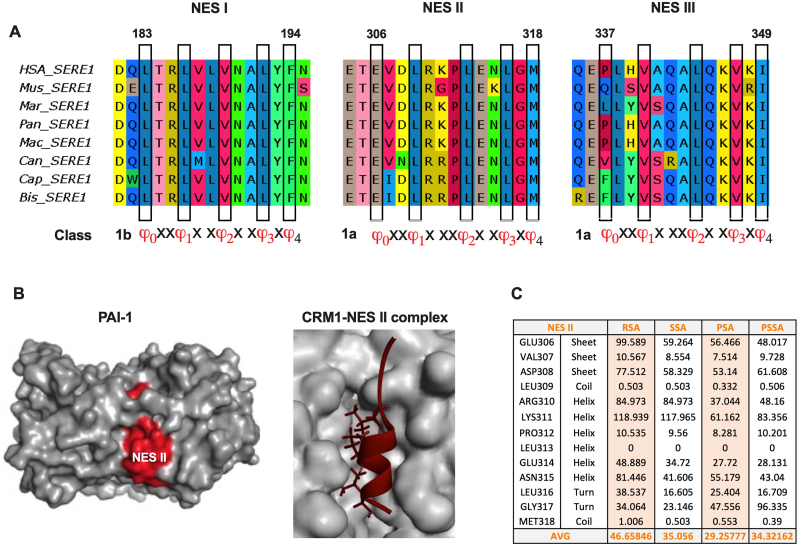
Identification of potential NES in PAI-1. A) The sequences of the NES motifs (NES I, NES II, NES III) identified in PAI-1 from selected vertebrate species were aligned. The specific class of consensus pattern in each NES motif is shown here. The conserved hydrophobic amino acids are shown in boxes. B) Left panel: The cartoon shows the surface model of PAI-1 (grey), and NES II is shown in red; Right panel: CRM1 bound with NES II motif. NES II motif is shown in red with hydrophobic residues as sticks, and the hydrophobic pocket of CRM1 is shown in grey. C) The table shows the average RSA, SSA, PSA, and PSSA of NES II. The PSA score >25 denotes that the NES is surface-exposed. AVG - average, RSA-residual solvent accessibility, SSA-sidechain solvent accessibility, PSA-percentage solvent accessibility, PSSA-percentage sidechain solvent accessibility. (For interpretation of the references to colour in this figure legend, the reader is referred to the Web version of this article.)

The nuclear export of proteins is mainly governed by the classical CRM1-dependent NES. NESs are 10–15 hydrophobic amino acid-long residues with specific consensus patterns and are classified into various classes [28]. Here, we used various bioinformatic tools to identify NES in human PAI-1, then manually curated the identified NES motifs. We identified 5 putative NES motifs (score >0.1) by using the LocNES tool (Table S1), while only 1 NES motif was identified by the ELM server search (Table S1). These motifs were further curated manually, and those which did not fit the criteria of different NES motif classes were rejected (Table S1). We also manually identified 1 putative NES motif (Table S1) based on the known consensus patterns of NES motifs [28]. Finally, 3 NES motifs (NES I, NES II, and NES III) were selected, which were highly conserved across different vertebrate species (Table S2, Fig. 1A–and S1), indicating that these motifs might be functional.

It has been shown that the NES motifs should follow consensus patterns, possess solvent-accessibility, and have a helical/coiled secondary structure [28]. To characterize the identified NESs further, the structure of human PAI-1 (PDB ID:1LJ5) was analyzed for the secondary structure and surface accessibility of NES motifs. NES I (aa 183–195) and NES III (aa 337–349) were buried in the PAI-1 structure (Fig. S2A) with PSA <25 (Fig. S2B), indicating that they are not accessible to the protein surface. Moreover, these NESs showed a beta sheet-like structure (Fig. S2B) that is not a typical conformation of NES motifs. Hence, these motifs were rejected. NES II (aa 306–318) acquired mainly a helix-like conformation, with PSA >25, indicating that it is accessible to the surface (Figs. S2 and 1B left panel, and 1C). NES II motif fits into the NES binding pocket of CRM1 protein (Fig. 1B right panel). These data showed that PAI-1 has 1 functional CRM1-dependent NES (NES II), and thus might shuttle between the nucleus and the cytoplasm.

Nuclear proteins contain nuclear localization signal (NLS) that are basic amino acid-rich motifs [29]. We asked whether PAI-1 has potential NLS motifs. To identify the NLS in PAI-1, we searched for basic amino acids (R and K) clusters in the PAI-1 protein sequence. We found that basic amino acid clusters (≥4 amino acids) were absent in PAI-1 (Fig. S3), indicating that PAI-1 contains no functional NLS. These findings suggest that PAI-1 might be exported from the nucleus using CRM1-dependent NES and enter the nucleus by interacting with an NLS-containing protein.

### 3.2 PAI-1 is localized in the nucleus of endothelial cells, and it is exported out in a CRM1-dependent manner

**Fig. 2:**
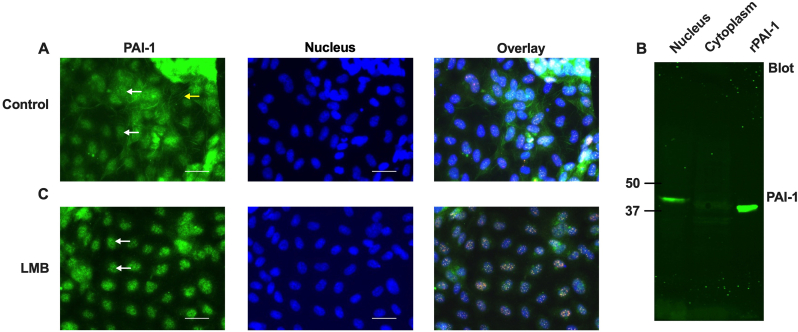
Subcellular distribution of PAI-1 in endothelial cells. A) HUVECs were stained with specific PAI-1 antibody (left panel, green), nuclei were stained with Hoechst 33258 (middle panel, blue) and overlay picture (right panel). PAI-1 was distributed in the cytoplasm (left panel, yellow arrow) and the nucleus (left panel, white arrows). The scale bar is 20 μm. B) Nuclear and cytoplasmic fractions of HUVECs were immunoblotted with PAI-1 antibody. Recombinant PAI-1 (rPAI-1) was used as control. C) The HUVECs were treated with LMB to block the nuclear export. PAI-1 was enriched in the nucleus after LMB treatment (left panel, white arrows). The scale bar is 20 μm. (For interpretation of the references to colour in this figure legend, the reader is referred to the Web version of this article.)

We wondered whether PAI-1 is present intracellularly in endothelial cells, and, if yes, whether it is in the nucleus of these cells. To explore the subcellular distribution of PAI-1 in endothelial cells, HUVECs were stained with specific PAI-1 antibody and observed under the fluorescence microscope. We found that PAI-1 was distributed in the nucleus (Fig. 2A left panel, white arrows) and the cytoplasm (Fig. 2A left panel, yellow arrow). To further confirm its localization in the nucleus, the nuclei of these cells were isolated and subjected to immunoblotting with a specific PAI-1 antibody. The purity of the nucleus was checked under the microscope after nuclear staining with Hoechst 33258 (Fig. S4). Also, the nuclear lysate was blotted with cytoplasmic marker tubulin antibody to confirm the purity of the nuclear fraction. No tubulin bands were observed in the nuclear fraction, confirming that it was free from cytoplasmic contamination (Fig. S4). We found a specific band of ∼45 kDa in the nuclear lysate of HUVECs, comparable to that of the recombinant PAI-1 (rPAI-1). A faint band was also observed in the cytoplasmic fraction (Fig. 2B). These data confirm that PAI-1 is localized intracellularly and distributed mainly in the nucleus of HUVECs.

To confirm whether the identified CRM1-specific NES in PAI-1 is functional and responsible for PAI-1 export from the nucleus, we treated HUVECs with a specific inhibitor of CRM1 protein; LMB, to block the CRM1-dependent nuclear export of PAI-1 [30]. Indeed, PAI-1 protein was enriched in the nucleus of HUVECs after blocking the CRM1-dependent nuclear export by LMB (Fig. 2C left panel, white arrows). We observed enriched dot-like structures in the Hoechst 33258-stained nuclei after LMB treatment (Fig. 2C middle panel). Interestingly, these dots were positively stained with PAI-1 antibody (Fig. 2C right panel), suggesting that PAI-1 was also enriched in these dots. These data indicate that PAI-1 has a functional CRM1-dependent NES motif that regulates the localization of PAI-1 in the nucleus. Moreover, PAI-1 may be localized in specific nuclear bodies or domains (dots) in the HUVECs.

### 3.3 PAI-1 is localized in PML bodies of the nucleus of endothelial cells

**Fig. 3:**
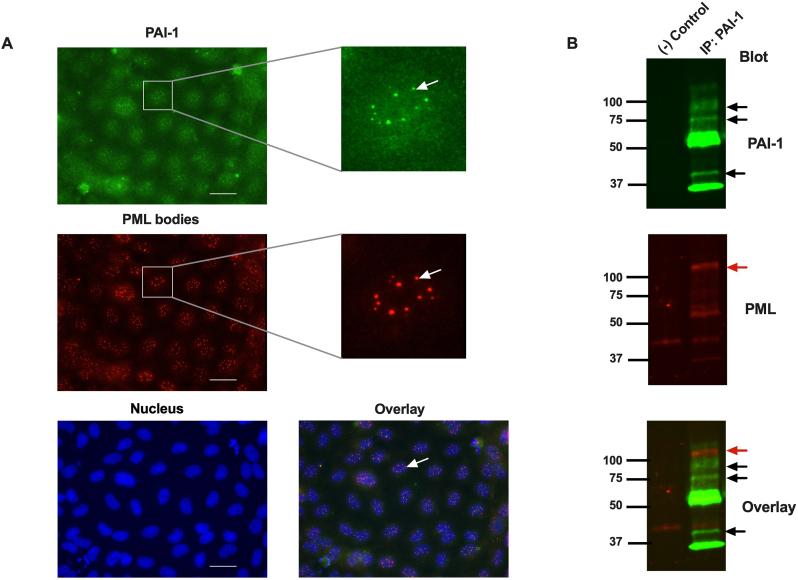
PAI-1 is localized in PML bodies of endothelial cells. A) HUVECs were stained with a specific PAI-1 antibody (upper left panel, green), which shows PAI-1 as dot-like structures in the nucleus (upper right panel, white arrow), and a specific PML antibody (middle left panel, red), highlighting dot-like structures of PML bodies (middle right panel, white arrow). The nuclei were stained with Hoechst 33258 dye (lower left panel, blue), and an overlay image shows the localization of PAI-1 in PML bodies (lower right panel, white arrow). The scale bar is 20 μm. B) The nuclei of HUVECs were isolated, and then PAI-1 was immunoprecipitated. Western blot was performed using antibodies against PAI-1 (upper panel, green) and PML (middle panel, red). A merged image with bands of both PAI-1 and PML is shown in the lower panel. Black arrows: PAI-1, red arrows: PML. (−) Control: negative control. (For interpretation of the references to colour in this figure legend, the reader is referred to the Web version of this article.)

Various nuclear bodies or domains with specialized functions are reported in the nucleus of eukaryotic cells [22]. PML bodies are dot-like nuclear bodies with a size ranging from 0.2 to 1 μm in diameter and mainly composed of PML protein [22]. We hypothesized that PAI-1 containing dot-like structures might be PML bodies. To explore the localization of PAI-1 in the PML bodies, the HUVECs were stained with specific PML and PAI-1 antibodies. Indeed, dot-like nuclear bodies in the nucleus of HUVECs were stained with the PML antibody, confirming that these dots are PML bodies (Fig. 3A middle and lower right panels, white arrows) and also were stained with PAI-1 antibodies (Fig. 3A, upper and lower right panel, white arrow). These data showed that PAI-1 is mainly localized in the PML bodies inside the nucleus of HUVECs.

To further confirm the localization of PAI-1 in the PML bodies, we immunoprecipitated PAI-1 from the nuclear lysate of HUVECs using a specific PAI-1 antibody. Indeed, PML protein was co-immunoprecipitated with PAI-1 (Fig. 3B), suggesting that PAI-1 is mainly localized in the PML bodies due to the interaction with PML protein. These data confirm that PAI-1 is imported to PML bodies in the nucleus due to the interaction of PAI-1 with PML protein.

### 3.4 Localization of PAI-1 in PML bodies is dependent on endothelial cell growth

**Fig. 4:**
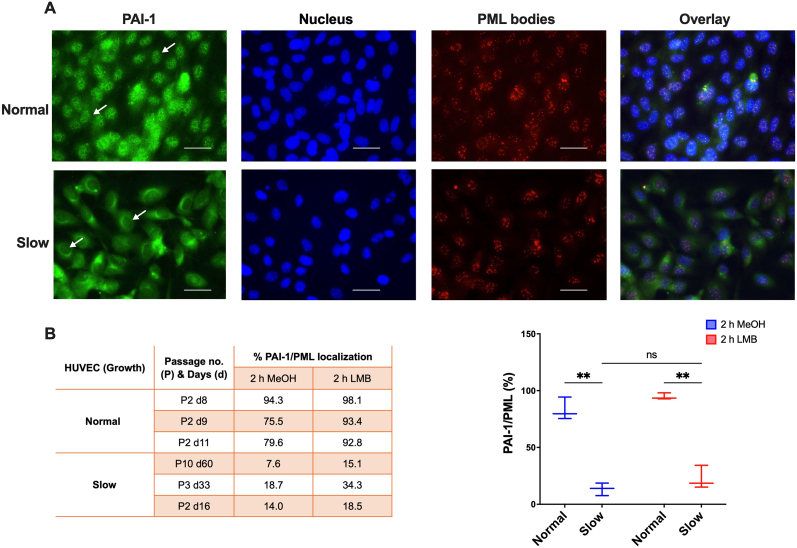
PAI-1 is enriched in PML bodies in a growth-dependent manner. A) Fast-growing (Normal) and slow-growing (Slow) HUVECs were stained with a PAI-1 antibody (first panel, green), the nuclei stained with Hoechst 33258 dye (second panel, blue), PML antibody (third panel, red), and the overlay picture (fourth panel). In contrast to “Normal” HUVECs, PAI-1 is mainly localized in the cytoplasm and not in the nucleus of "Slow" HUVECs. The scale bar is 20 μm. B) The “Normal” and “Slow” HUVECs were treated with a CRM1-specific inhibitor LMB. Left panel: The table shows the percentage localization of PAI-1 in PML bodies of “Normal” and “Slow” HUVECs. Right panel: The graph shows the distribution of PAI-1 with respect to PML in “Normal” and “Slow” HUVECs before and after LMB treatment.**p *<* 0.01, ns: not significant. (For interpretation of the references to colour in this figure legend, the reader is referred to the Web version of this article.)

During this study, we observed that the growth of HUVECs isolated from the umbilical cords of different donors and grown up to 80 % confluence, varied drastically after splitting them in a 1:5 ratio. Cells from some donors reached 80 % confluence again within 5–7 days of passaging; while those from others could not attain it even after two weeks. In this direction, we categorized HUVECs as “Normal” (fast-growing) and “Slow” (slow-growing), wherein the cells that were in culture for 8–11 days, and fast-growing were considered as “Normal,” while those that were in culture for 16–60 days and grew slowly were termed as “Slow”. Notably, we also observed that the localization of PAI-1 in the PML bodies was inconsistent and highly dependent on the growth properties of HUVECs. We then asked whether PAI-1 localization in PML bodies is correlated with the growth potential of endothelial cells. We found that the endogenous PAI-1 is localized in the PML bodies inside the nucleus of “Normal” cells (Fig. 4A upper panel). Contrary to this, we could hardly detect PAI-1 localization in the nucleus and PML bodies in “Slow” endothelial cells (Fig. 4A lower panel).

Furthermore, we quantified the localization of PAI-1 in PML bodies, which was on average, 83 % in “Normal” HUVECs, whereas, in slow-growing HUVECs, it was 13 % (Fig. 4B left panel). Interestingly, the nuclear expression of PAI-1 gets enriched in “Normal” HUVECs upon LMB treatment in comparison to the control MeOH-treated cells (Fig. 4B left panel), while the enrichment is not significant in the “Slow” cells, and was found comparable to the control cells (Fig. 4B), indicating that PAI-1 was mainly restricted in the cytoplasm, and did not enter the nucleus in “Slow” cells. Therefore, we anticipate that PAI-1 might have an age-related function in the nucleus of endothelial cells.

## 4 Discussion

PAI-1, a secretory protein, is an inhibitor of fibrinolysis and is involved in aging-associated thrombosis and other pathologies [31]. It has a paradoxical role in tumor progression, but its molecular functions are poorly understood. Here, we identified an unconventional distribution of PAI-1 in the PML bodies of the nucleus of endothelial cells. We demonstrated that PAI-1 has a functional CRM1-specific NES. We showed that the localization of PAI-1 in PML bodies is highly correlated with the growth potential of endothelial cells. Together, this study identifies a novel behavior of PAI-1 and may be able to explain its paradoxical role in cancer.

Vertebrate serpins mainly function as extracellular protease inhibitors; only the Clade B members lack signal peptides and are localized inside the cells [1]. PAI-1 is a profound Clade E (V3 group) member of the Serpin superfamily and is secreted from the cell [1]. Previous studies indicate the intracellular presence of PAI-1 [25,26]. However, based on the present knowledge, it is unlikely that PAI-1 can localize in the nucleus. Still, we cannot rule out its nuclear localization under specific cellular states or pathophysiological conditions. This contrasting information inspired us to explore whether PAI-1 localizes in the nucleus.

The active nucleocytoplasmic shuttling of any cargo protein is governed by specific amino acid sequences: basic amino acid-rich NLS and hydrophobic amino acid-rich NES. We could not identify any NLS in PAI-1, suggesting that PAI-1 may interact with an NLS-containing protein to enter the nucleus. It is unlikely that PAI-1 passively diffuses in the nucleus due to its molecular weight (∼40 kDa), and the presence of signal sequence [[32], [33], [34]]. A study showed that intracellular serpin PI-9, which has a similar molecular weight and does not possess an NLS, is actively imported into the nucleus [33]. It supports our hypothesis that PAI-1 actively imports into the nucleus of endothelial cells.

Classical CRM1-dependent NES motifs [30] are 10–15 hydrophobic amino acid-long residues (mainly leucine-rich) that are spaced with a specific consensus pattern (Φ_0_xxΦ_1_x_(2,3)_Φ_2_ x_(2,3)_Φ_3_xΦ_4_, where x is any amino acid, and Φ are hydrophobic amino acids), and are categorized into various classes [28,35]. The NES motifs should follow consensus patterns, be solvent-accessible, and possess a helical/coiled secondary structure [28]. We identified a potential CRM1-dependent NES (NES II) in PAI-1 that might be responsible for its export from the nucleus. Indeed, PAI-1 was enriched in the nucleus of endothelial cells after treatment with CRM1-specific inhibitor; LMB. In this study, we showed, for the first time, that PAI-1 is not only an extracellular protein, but also actively shuttles between the nucleus and cytoplasm via a CRM1-dependent NES.

Interestingly, we observed punctate structures of PAI-1 in the nucleus of HUVECs, indicating that PAI-1 might be present in specific nuclear bodies. Indeed, PAI-1 localizes with PML nuclear bodies, suggesting that PAI-1 might interact with PML protein and get translocated to PML bodies in the nucleus.

Previous studies suggest that PML bodies are involved in various cellular functions, including cell apoptosis and senescence [36,37], and that PAI-1 is a critical regulator of senescence, but its precise role is poorly understood. We observed a substantial variation in the growth potential of isolated HUVECs depending on the quality of the umbilical cord and the cell culture conditions. Interestingly, the localization of PAI-1 in the PML bodies is not uniform, and it is dominant in the “Normal” and proliferative cells. The localization was sparsely observed in “Slow” and non-proliferative cells. We suggest that PAI-1 actively shuttles between the nucleus and the cytoplasm in “Normal” cells, while in “Slow” cells, the localization of PAI-1 was restricted in the cytoplasm even upon LMB treatment, suggesting that the nuclear import of PAI-1 is blocked in “Slow” endothelial cells. These data corroborate that the localization of PAI-1 in PML bodies might be required to control the proliferation of the endothelial cells.

## 5 Conclusion

In this study, we showed, for the first time, that the localization of PAI-1 in PML bodies is highly correlated with the growth potential of endothelial cells and could impart a strong link to its age-related functions and tumor progression. The pro-tumorigenic role of PAI-1 could be due to its function in the PML bodies, and the anti-tumorigenic property may be due to the extracellular PAI-1 function as a protease inhibitor. Together, this study identifies a novel behavior of PAI-1 that might be linked with cell aging and may be able to answer the elusive role of PAI-1 in tumor progression.

## CRediT authorship contribution statement

**Pragya Gehlot:** Writing – review & editing, Writing – original draft, Visualization, Validation, Investigation, Data curation, Conceptualization. **Daniela Brünnert:** Writing – review & editing, Writing – original draft, Validation, Investigation, Formal analysis, Data curation, Conceptualization. **Vibha Kaushik:** Writing – review & editing, Writing – original draft, Visualization, Investigation. **Arpana Yadav:** Writing – review & editing, Visualization, Investigation. **Saloni Bage:** Writing – review & editing, Investigation. **Kritika Gaur:** Visualization, Investigation. **Mahesh Saini:** Writing – original draft, Investigation. **Jens Ehrhardt:** Investigation. **Gowrang Kasaba Manjunath:** Investigation. **Abhishek Kumar:** Writing – review & editing, Investigation. **Neena Kasliwal:** Writing – review & editing, Writing – original draft, Visualization. **Ajay Kumar Sharma:** Writing – review & editing, Investigation. **Marek Zygmunt:** Writing – review & editing, Resources, Funding acquisition. **Pankaj Goyal:** Writing – review & editing, Writing – original draft, Visualization, Validation, Supervision, Resources, Project administration, Investigation, Funding acquisition, Formal analysis, Data curation, Conceptualization.

## Declaration of competing interest

The authors declare that they have no known competing financial interests or personal relationships that could have appeared to influence the work reported in this paper.
