# Supplementary material for: Unconventional localization of PAI-1 in PML bodies: A possible link with cellular growth of endothelial cells
Source: Biochem Biophys Rep. 2024 Jul 26;39:101793. doi: 10.1016/j.bbrep.2024.101793 (PMC11332193; doi:10.1016/j.bbrep.2024.101793)
Supplement: Multimedia component 1 [file mmc1.pdf]

Unconventional localization of PAI-1 in PML bodies: a possible link with cellular growth of endothelial cells

Pragya Gehlot[1#], Daniela Brünnert[*2,3#], Vibha Kaushik[1], Arpana Yadav[1], Saloni Bage[1], Kritika Gaur[1], Mahesh Saini[1], Jens Ehrhardt[3], Gowrang Kasaba Manjunath[4,5], Abhishek Kumar[4,5], Neena Kasliwal[6], Ajay Kumar Sharma[7], Marek Zygmunt[3], and Pankaj Goyal[*1]

[1]Department of Biotechnology, School of Life Sciences, Central University of Rajasthan, Bandarsindri, Kishangarh 305 817, Rajasthan, India

[2]University Hospital of Würzburg, Department of Obstetrics and Gynecology, Josef-Schneider-Str. 4, D-97080, Würzburg, Germany

[3]Department of Obstetrics and Gynecology, University of Greifswald, Ferdinand-Sauerbruchstrasse, D-17489, Greifswald, Germany

[4]Manipal Academy of Higher Education (MAHE), Manipal 576104, Karnataka, India

[5]Institute of Bioinformatics, International Technology Park, Bangalore 560066, Karnataka, India

[6]Department of Pathology, J.L.N. Medical College, Ajmer 305001, Rajasthan, India

[7]Department of Obstetrics and Gynecology, J.L.N. Medical College, Ajmer 305001, Rajasthan, India

Table S1: Prediction of NES motifs based on primary sequence analysis. LocNES tool, ELM database, and manual curation method were used to identify putative NES motifs in the primary amino acid sequence of PAI-1 (NP_000593.1). Hydrophobic amino acids are shown in red, and proline is highlighted in yellow.

Tool/Database	Putative NES motifs			Consensus Class
	Position	Sequence	Score	
LocNES	1-18	MQMSPALTCLVLGLALVF	0.664	Rejected as it is a signal peptide
	179-194	AVDQLTRLVLVNALYF	0.238	1b
	292-303	LPRLLVLPKAAAFSL	0.111	Rejected as proline is not allowed at Φ1 and Φ2
	304-318	ETEVDLRKPLENLGM	0.423	1a
	318-332	MTDMFRQFQADFTSL	0.102	No matched consensus class
ELM server	306-318	EVDLRKPLENLGM	NA	1a
Manual curation	335-349	QEPLHVAQALQKVKI	NA	1a

Table S2: List of the accession numbers of PAI-1 protein sequences from the selected vertebrate species used in this study.

Organism	Common name	Protein code	NCBI ID
Homo sapiens	Human	HSA_SERE1	NP_000593.1
Chelonoidis niger abingdonii	Pinta giant tortoise	Che_SERE1	XP_032622720.1
Mus musculus	Mouse	Mus_SERE1	NP_032897.2
Ochotona princeps	American pika	Och _SERE1	XP_004587240.2
Equus caballus	Horse	Equ _SERE1	AAM34252.1
Marmota marmota marmota	Alpine marmot	Mar_SERE1	XP_015346959.1
Loxodonta africana	African savanna elephant	Lox_SERE1	XP_003416577.1
Pan troglodytes	Chimpanzee	Pan_SERE1	XP_527841.4
Macaca mulatta	Rhesus monkey	Mac_SERE1	XP_001107647.1
Canis lupus familiaris	Dog	Can_SERE1	NP_001184024.1
Capra aegagrus hircus	Goat	Cap_SERE1	XP_005701338.1
Bison bison bison	American bison	Bis_SERE1	XP_010830894.1
Camelus dromedarius	Arabian camel	Cam_SERE1	KAB1263232.1
Delphinapterus leucas	Beluga whale	Del_SERE1	XP_022444348.1
Danio rerio	Zebra fish	Dan_SERE1	NP_001108031.1
Scleropages formosus	Asian arowana fish	Scl_SERE1	XP_018588767.1

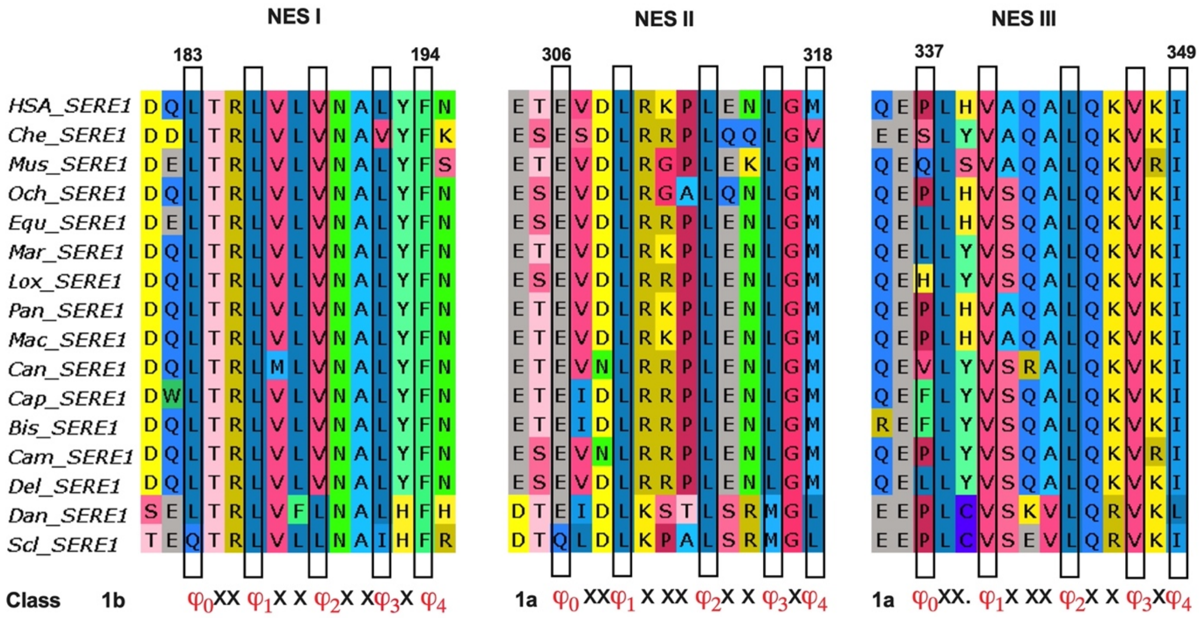

Figure S1: The multiple sequence alignment of NES motifs from selected vertebrate species. The sequences of the three NES motifs (NES I, NES II, NES III) identified in PAI-1 from selected species of vertebrates were aligned. The specific class of consensus pattern in each NES motif is shown here. The conserved hydrophobic amino acids at φ positions are shown in boxes.

A

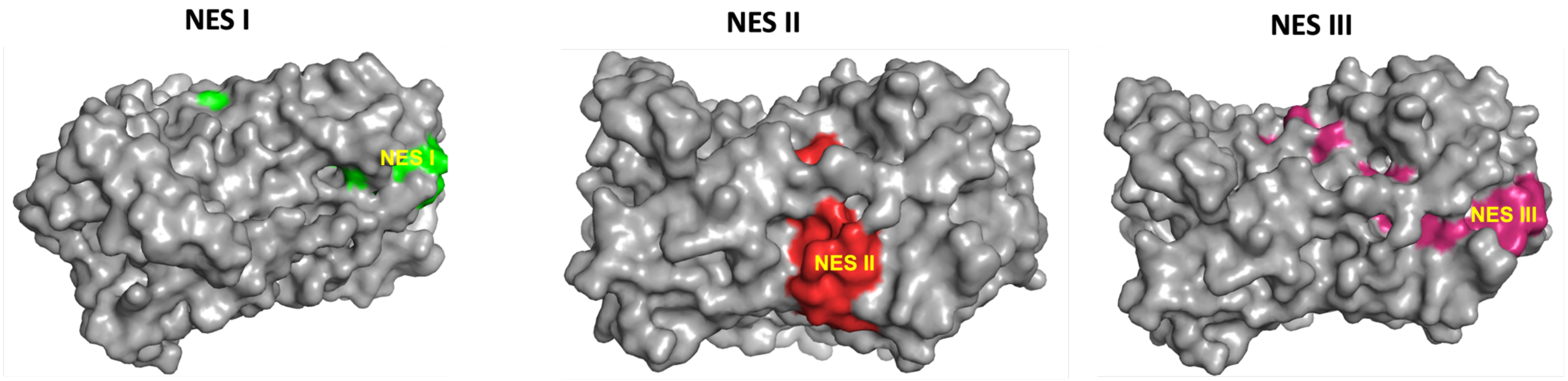

B

NES I		RSA	SSA	PSA	PSSA
LEU183	Turn	143.829	126.298	94.815	127.089
THR184	Coil	6.19	4.712	4.668	5.462
ARG185	Coil	71.077	68.155	30.986	38.628
LEU186	Sheet	0	0	0	0
VAL187	Sheet	0	0	0	0
LEU188	Sheet	2.516	2.516	1.659	2.532
VAL189	Sheet	0	0	0	0
ASN190	Sheet	0	0	0	0
ALA191	Sheet	0	0	0	0
LEU192	Sheet	2.013	2.013	1.327	2.025
TYR193	Sheet	2.859	2.859	1.302	1.697
PHE194	Sheet	0	0	0	0
AVG		19.04033	17.21275	11.22975	14.78608

NES II		RSA	SSA	PSA	PSSA
GLU306	Sheet	99.589	59.264	56.466	48.017
VAL307	Sheet	10.567	8.554	7.514	9.728
ASP308	Sheet	77.512	58.329	53.14	61.608
LEU309	Coil	0.503	0.503	0.332	0.506
ARG310	Helix	84.973	84.973	37.044	48.16
LYS311	Helix	118.939	117.965	61.162	83.356
PRO312	Helix	10.535	9.56	8.281	10.201
LEU313	Helix	0	0	0	0
GLU314	Helix	48.889	34.72	27.72	28.131
ASN315	Helix	81.446	41.606	55.179	43.04
LEU316	Turn	38.537	16.605	25.404	16.709
GLY317	Turn	34.064	23.146	47.556	96.335
MET318	Coil	1.006	0.503	0.553	0.39
AVG		46.65846	35.056	29.25777	34.32162

NES III		RSA	SSA	PSA	PSSA
PRO337	Coil	94.741	73.464	74.477	78.389
LEU338	Coil	2.955	0.503	1.948	0.506
HIS339	Coil	44.353	42.923	25.335	34.579
VAL340	Coil	1.006	1.006	0.716	1.144
ALA341	Coil	26.685	10.567	26.653	22.951
GLN342	Sheet	40.677	39.671	22.814	31.646
ALA343	Sheet	2.468	1.006	2.465	2.186
LEU344	Sheet	10.567	10.567	6.966	10.633
GLN345	Sheet	0.471	0.471	0.264	0.376
LYS346	Sheet	17.112	17.112	8.8	12.092
VAL347	Sheet	0	0	0	0
LYS348	Sheet	33.347	33.347	17.148	23.563
ILE349	Sheet	0	0	0	0
AVG		21.10631	17.74131	14.42969	16.77423

Figure S2: Surface accessibility of putative NESs identified in PAI-1. A) The surface of human PAI-1 is shown in grey, and NES I (left panel, green), NES II (middle panel, red), and NES III (right panel, pink) are shown. B) The tables show the average RSA, SSA, PSA, and PSSA of the three NES motifs. The PSA score > 25 denotes that the NES is surface-exposed. AVG- average, RSA- residual solvent accessibility, SSA- sidechain solvent accessibility, PSA- percentage solvent accessibility, PSSA- percentage sidechain solvent accessibility.

```
         10        20        30        40        50
          |         |         |         |         |
MQMSPALTCLVLGLALVFGEGSAVHHPPSYVAHLASDFGVRVFQQVAQAS
KDRNVVFSPYGVASVLAMLQLTTGGETQQQIQAAMGFKIDDKGMAPALRH
LYKELMGPWNKDEISTTDAIFVQRDLKLVQGFMPHFFRLFRSTVKQVDFS
EVERARFIINDWVKTHTKGMISNLLGKGAVDQLTRLVLVNALYFNGQWKT
PFPDSSTHRRLFHKSDGSTVSVPMMAQTNKFNYTEFTTPDGHYYDILELP
YHGDTLSMFIAAPYEKEVPLSALTNILSAQLISHWKGNMTRLPRLLVLPK
FSLETEVDLRKPLENLGMTDMFRQFQADFTSLSDQEPLHVAQALQKVKIE
VNESGTVASSSTAVIVSARMAPEEIIMDRPFLFVVRHNPTGTVLFMGQVM
EP
```

Figure S3: The primary sequence of PAI-1 highlighting the basic amino acids in red.

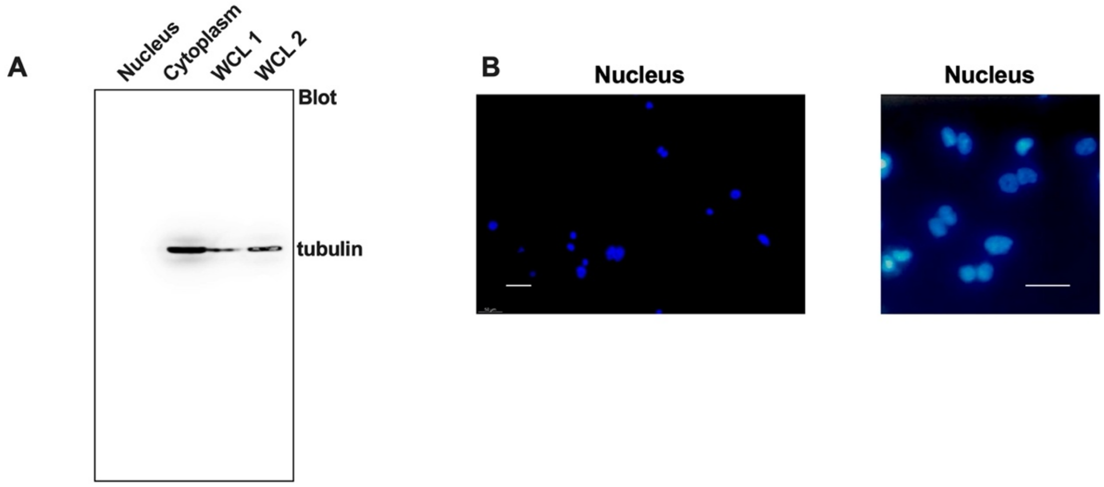

Figure S4: Purity of nuclear fractions. The nuclei were isolated from HUVECs, and the purity was determined by Western blotting and fluorescence microscopy using the nuclear staining dye Hoechst 33258. A) Different cellular fractions of HUVECs were blotted with cytoplasmic marker tubulin antibody. No tubulin band was observed in nuclear fraction indicating that the isolated nuclei were free from cytoplasmic contamination. A prominent band of tubulin was observed in cytoplasmic fraction and whole cell lysates (WCL 1 and WCL 2). B) The purified nuclei were stained with Hoechst 33258 dye which specifically stained the nucleus. The fluorescent images show the intact blue nuclei confirming the purity of the isolated nuclei. Scale bar is 50 µm.
